# Feline Heartworm in Clinical Settings in a High Canine Prevalence Area

**DOI:** 10.3389/fvets.2022.819082

**Published:** 2022-02-10

**Authors:** Bruno Alberigi, Diefrey Ribeiro Campos, Aline Serricella Branco, Alexandre Bendas, Rodrigo Pereira Brum, Raquel Calixto, Leucio Câmara Alves, Jose Wilton Pinheiro Júnior, Fabiana Batalha Knackfuss, Norma Labarthe, Julie K. Levy, Flavya Mendes-de-Almeida

**Affiliations:** ^1^Departamento de Medicina e Cirurgia Veterinária, Instituto de Veterinária, Universidade Federal Rural do Rio de Janeiro (Federal Rural University of Rio de Janeiro), Seropédica, Brazil; ^2^Programa de Pós-graduação em Ciências Veterinárias, Departamento de Parasitologia Animal, Instituto de Veterinária, Universidade Federal Rural do Rio de Janeiro (Federal Rural University of Rio de Janeiro), Seropédica, Brazil; ^3^PET CARE Animalia, Rio de Janeiro, Brazil; ^4^Laboratório de Doenças Parasitárias, Departamento de Medicina Veterinária, Universidade Federal Rural de Pernambuco, Recife, Brazil; ^5^Curso de Medicina Veterinária, Universidade do Grande Rio, Duque de Caxias, Brazil; ^6^Programa de Pós-graduação em Ética, Bioética e Saúde Coletiva, Fundação Oswaldo Cruz, Rio de Janeiro, Brazil; ^7^Maddie's Shelter Medicine Program, College of Veterinary Medicine, University of Florida, Gainesville, FL, United States; ^8^Departamento de Patologia e Clínica Veterinária, Faculdade de Veterinária, Universidade Federal Fluminense, Niterói, Brazil

**Keywords:** heartworm disease, clinical signs, feline immunodeficiency virus, feline leukemia virus, cats

## Abstract

Heartworm (HTW) infection in cats is associated with persistent pulmonary pathology, even when clinical signs are absent. Treatment options for cats are limited once infected, making prevention an important topic for discussion with cat owners. In Brazil, tests to detect feline HTW infections are unavailable, likely leading to an underestimation of its impact on the wellbeing of cats. The present study investigated the seroprevalence of HTW antigen in cats living in an area with high canine HTW prevalence and investigated risk factors and clinical signs associated with HTW disease in cats from Rio de Janeiro, Brazil. Clinical examinations were conducted, and serological evaluations performed with a point-of-care test (SNAP^®^ Feline Triple^®^ Test, IDEXX Laboratories, Inc.). A total of 586 cats were examined. The HTW antigen seroprevalence was 1.2%. Heartworm positive results were significantly associated with vomiting and abnormal lung sounds. Results from this study indicate that cats residing in areas of high canine HTW prevalence should have HTW disease as a differential diagnosis when presenting with compatible clinical signs. Veterinarians should prioritize client education and promote regular use of effective prophylaxis to protect feline health.

## Introduction

*Dirofilaria immitis* is a mosquito-borne nematode well-adapted to canid hosts and the causative agent of heartworm (HTW) disease. In Brazil, canine HTW infections have been reported continuously over time in dogs from all geographical regions (1, 2) with a prevalence as high as 62% in some areas (3). Although less well-adapted to felids, *D. immitis* has been found to infect cats in regions where the infection is endemic in canids (4, 5). The lack of readily available point-of-care (POC) diagnostic tests to diagnose feline HTW infections in Brazil, either by antibody (Ab) or antigen (Ag), suggests that feline HTW infections are likely underrecognized relative to canine HTW. Additionally, many cats with *D. immitis* infections are asymptomatic, making it difficult for the veterinarian to suspect infection (6).

As in the dog, *D. immitis* infection in the cat begins with the bite of an infected mosquito and transmission of infective L3 larvae. However, in the cat, fewer larvae survive the molt to L4s and adults than they do in dogs (7), resulting in a lower mature worm burden in cats compared to dogs (8). When immature forms die during migration or when they arrive in the lungs, they elicit an acute pulmonary inflammatory response characterizing heartworm-associated respiratory disease (HARD) (7). The pulmonary inflammation elicited leads to clinical signs such as coughing, respiratory distress, vomiting, and sudden death (7, 9). These signs may appear as early as 3 months after infection and may be misdiagnosed as asthma (7, 10). The diagnosis of HARD can be improved by the addition of HTW-Ab detection because the detection of HTW-Ag is currently only possible after mature, adult parasites are present (6).

Treatment of *D. immitis* infection in the cat also differs from that of the dog. While dogs with HTW disease are to undergo adulticidal treatment with arsenical drugs approved for this use, adulticidal treatment is avoided in cats due to toxicity and adverse outcomes when adult worms die (6, 7, 11). When cats develop HTW disease they can only receive supportive therapy to help control clinical signs (6). Preventing heartworm infections is therefore important for cats and has been found to be highly effective (12). Cats living in endemic areas should be screened by HTW-Ag testing before implementing chemoprophylaxis. Cats presenting with respiratory signs or unexplained vomiting should also be tested; with the option of screening for the presence of HTW-Ab if Ag results are negative (10).

The present study investigated the seroprevalence of HTW antigen in cats living in an area with high canine HTW prevalence and investigated risk factors and clinical signs associated with HTW disease in cats from Rio de Janeiro, Brazil.

## Materials and Methods

### Animals

Inclusion criteria were defined as cats over 12 months of age, living in the metropolitan area of Rio de Janeiro, regardless of sex or breed. Cats were considered as adults (up to 6 years of age) or mature/senior (over 6 years of age) (13). Cat owners were invited to provide written consent to the inclusion of their cat in the study upon presenting to one of the study veterinarians. When owners had other cats, they were invited to bring them to be examined as long as they matched the inclusion criteria. When there were more than 10 cats to be examined, veterinarians visited the home at the owner's request. Animals from multi-cat households and animal shelters/sanctuaries were included according to the owner's discretion.

The housing locations were categorized as urban (3,000–10,000 hab/km^2^; >80% of dwelling-places with sanitary sewer) or suburban (275–2,800 hab/km^2^; <80% of dwelling-places with sanitary sewer and neighboring a large natural conservation area). The cat's type of ownership was recorded as household pet or shelter/sanctuary.

### Blood Samples

Blood samples were collected from the jugular, cephalic, or saphenous veins using sterile 23G intravenous butterfly needle and 3 mL syringes. The sample was immediately transferred to a preservative-free tube. When blood samples were collected at the clinic, serum was harvested in 10–20 min. When blood samples were collected outside the clinics, tubes were kept in coolers until they arrived at the clinics (<6 h), and serum was harvested immediately thereafter. Serum samples were stored at 4–6°C for maximum of 2 days and were tested for the presence of *D. immitis* antigen (HTW-Ag), feline immunodeficiency virus (FIV)-Ab and feline leukemia virus (FeLV)-Ag using SNAP^®^ Feline Triple^®^ Test (imported for research purposes by IDEXX Laboratories, São Paulo, Brazil, from IDEXX Laboratories, Westbrook, ME, USA) according to the manufacturer's instructions.

### Physical Examination and Patient History

A physical examination was carried out whenever the cat's behavior permitted. Body condition score (BCS) was evaluated from 1 to 9 (14) and clinical signs were explored according to routine clinical examination. Coughing, vomiting, and comorbidities were recorded from the information provided by the owner in response to a standardized patient enrollment questionnaire. The enrollment questionnaire also addressed heartworm risk factors, such as indoor/outdoor access and cohabitation with dogs. Information on heartworm chemoprophylaxis and FeLV vaccination was also obtained.

### Statistical Analysis

Analyses were carried out in Statistical Package for the Social Sciences SPSS software (IBM^®^), version 25.0. Univariate logistic regression was used, and odds ratios estimated to evaluate risk factors (breed, sex, reproductive status, age, outdoor access, household origin, cohabitation with dogs, heartworm chemoprophylaxis, FIV-Ab, FeLV-Ag) associated with the outcomes of interest and their 95% confidence intervals. A binomial random effects logistic regression (*P* < 0.05) model was used to identify associations between each of the clinical signs and detection of HTW-Ag.

## Results

**Figure 1:**
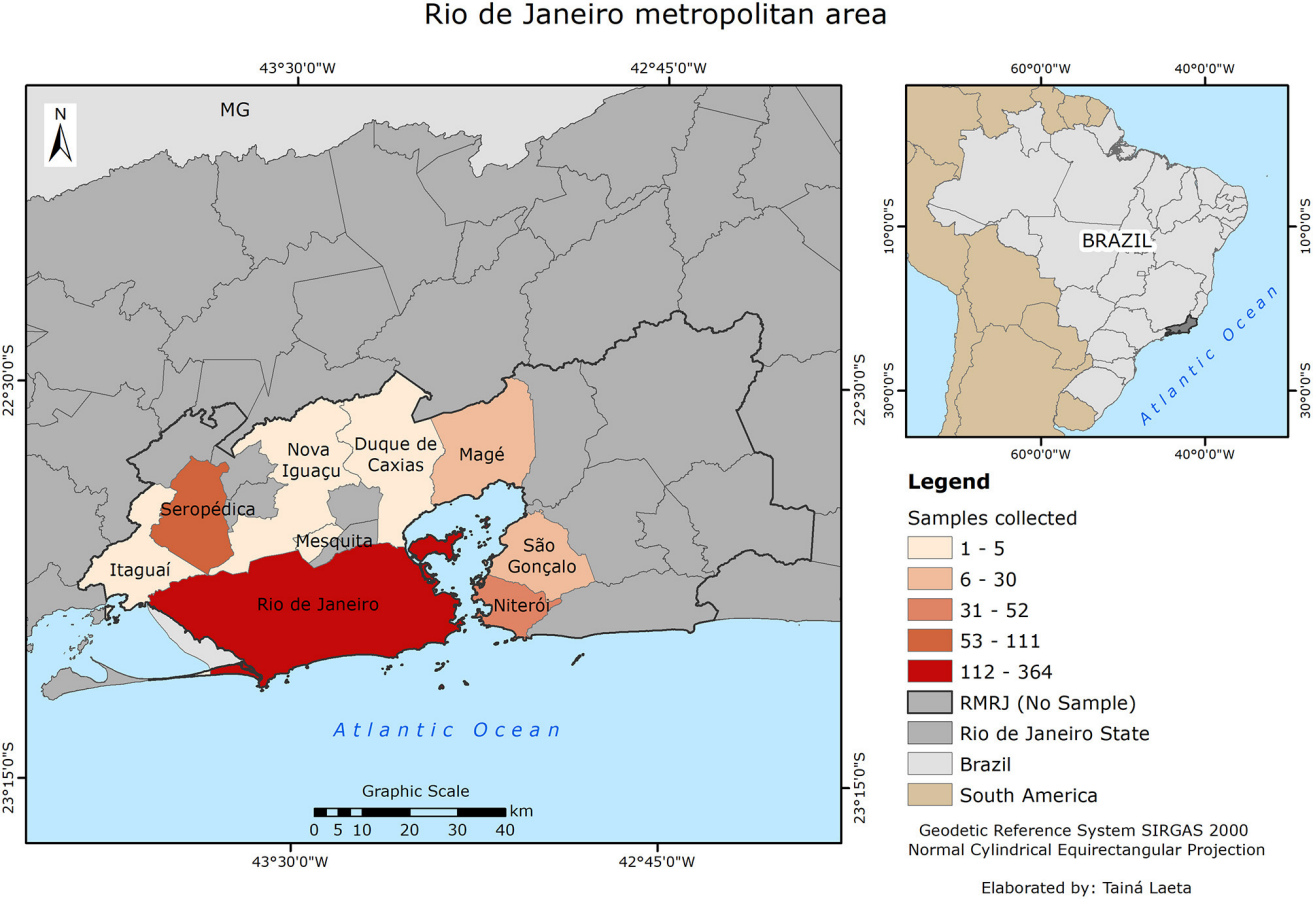
Map showing the municipalities of Rio de Janeiro metropolitan (RMRJ) area highlighting those where cats were examined according to the number of samples obtained.

A total of 586 cats from nine different municipalities in the metropolitan area of Rio de Janeiro were included (Figure 1). Most cats had outdoor access (82.6%; 484/586), including 76.9% of those from the urban area (143/186) and 85.3% from the suburban area (341/400). Most cats were female (59.7%; 350/586); adult (72.4%; 424/586); and neutered (91.5%; 536/586) (Table 1). The number of cats kept in shelter/sanctuary facilities (51%; 299/586) was similar to the number kept as household pets (49%; 287/586). Cats lived alone (5.1%; 30/586) or in groups categorized as <10 cats (33.4%; 196/586); 10–50 cats (31.9%; 187/586), and more than 50 cats (29.5%; 173/586).

**Table 1:** Potential risk factors for HTW-Ag seropositivity in cats in the Metropolitan region of Rio de Janeiro, Brazil.

**Risk factor**	***N***	**HTW-Ag**		
		***n*** **(%)**	**OR (95 %CI)**	***p*** **-value**
Total	586	7 (1.2)		
**Breed**				
Yes	42	1 (2.4)	–	0.99
No	544	6 (1.1)		
**Sex**				
Male	236	2 (0.9)	–	0.80
Female	350	5 (1.4)		
**Reproductive status**				
Intact	50	1 (0.02)	–	0.89
Neutered	536	6 (1.1)		
**Age group**				
Adult	424	5 (1.2)	–	0.71
Mature/senior	162	2 (1.2)		
**Outdoor access**				
Yes	484	6 (1.2)	–	0.78
No	102	1 (1.0)		
**Household origin**				
Urban	186	0	–	–
Suburban	400	7 (1.8)		
**Cohabitations with dogs**				
Yes	304	4 (1.3)	–	0.92
No	282	3 (1.0)		
**HTW chemoprophylaxis**				
Yes	71	0	–	–
No	515	7 (1.4)		
**FIV-Ab**				
Yes	28	0	–	–
No	558	7 (1.3)		
**FeLV-Ag**				
Yes	60	0	–	–
No	526	7(1.3)		

*HTW-Ag, Dirofilaria immitis antigen; FIV-Ab, feline immunodeficiency virus antibody; FeLV-Ag, feline leukemia virus antigen*.

*Univariate logistic regression was used, and odds ratios estimated to evaluate risk factors with 95% confidence intervals*.

Heartworm infection was detected in 1.2% of the cats (7/586), including one cat that was kept indoors only. Among the seven HTW infected cats, six received no HTW chemoprophylaxis and one had a history receiving <30% of the proper annual doses on a routine basis, therefore it was considered as incomplete HTW chemoprophylaxis. Of the cats receiving preventative medication on an annual basis according to label instructions (12.1%; 71/586), all were free from the infection (Table 1). All 158 cats vaccinated for FeLV had no FeLV-Ag detected when tested.

**Figure 2:**
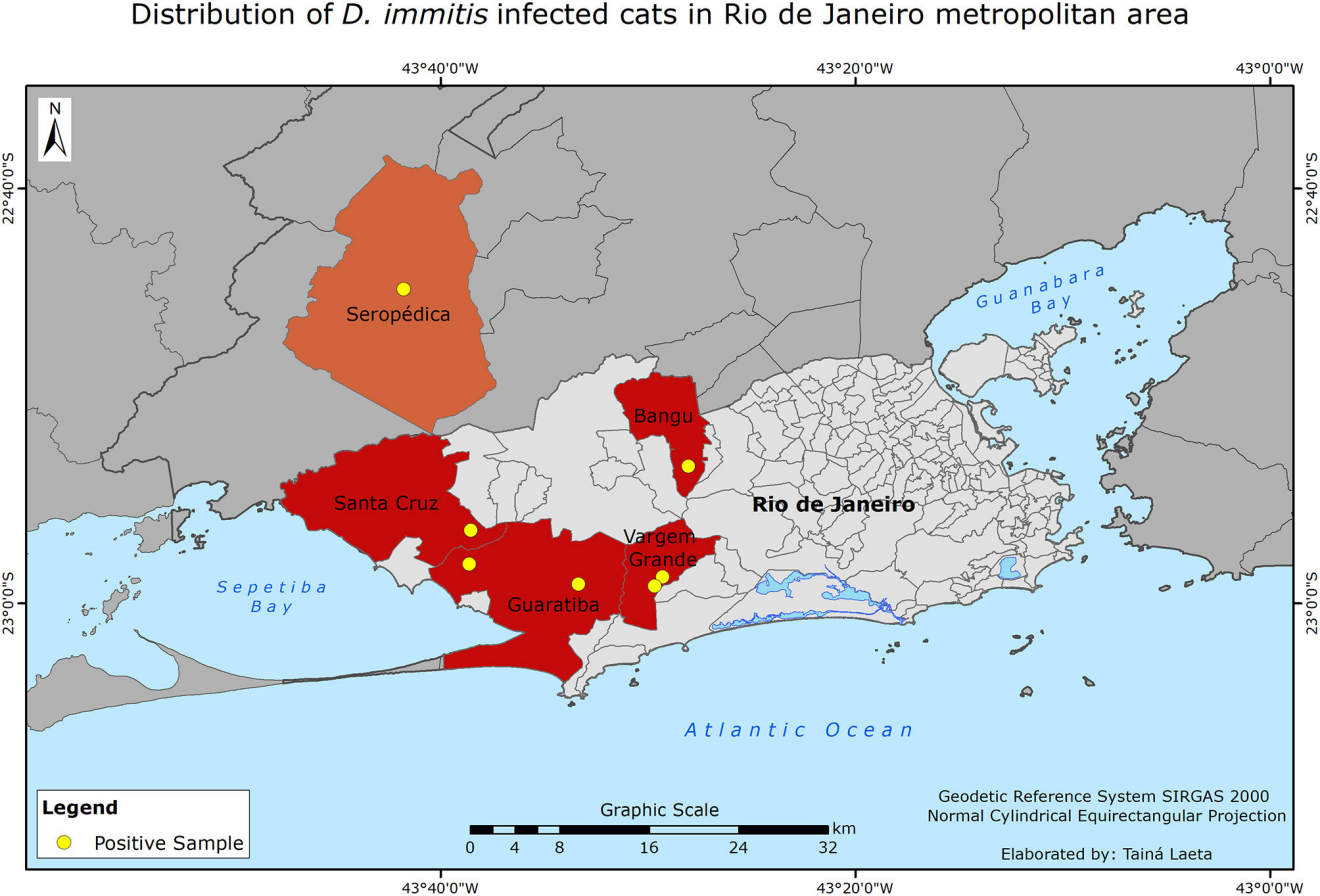
Map showing the locations where the *Dirofilaria immitis* infected cats lived.

None of the risk factors investigated was shown to enhance the likelihood of HTW infection, including the retrovirus infections (Table 1). The *D. immitis* infected cats were from suburban areas, six from the Western area of Rio de Janeiro municipality and one from Seropédica municipality (Figure 2). None of the cats with a positive HTW-Ag result tested positive for FIV-Ab or FeLV-Ag (Table 1). Two of the HTW infected cats lived in the same household with other 48 cats.

### Clinical Signs

The behavior of most (78.7%; 461/586) of the cats permitted a full physical evaluation. The number of cats with information on specific clinical signs varied because the owners were only able to supply complete information about their animals' clinical signs for 70.5% (413/586) of the cats. The only clinical sign found to be significantly associated with HTW-Ag was vomiting (*P* = 0.04). Although the association between the occurrence of heart murmur or irregular heart rhythm with the infection could not be demonstrated, it is noteworthy that all six heartworm infected cats that could be auscultated had heart murmurs (Table 2).

**Table 2:** Clinical signs associated with HTW-Ag seropositivity in cats in the Metropolitan region of Rio de Janeiro, Brazil.

		**HTW-Ag**	
	N^*^	**Positive** ***n*** **(%)**	***p*** **-value**
Body condition score^**^			
Too thin	44	1 (2.3)	–
Ideal/above ideal	387	6 (1.6)	
Overweight/obese	155	0	
**Dyspnea**			
Absent	569	6 (1.1)	0.52
Present	17	1 (5.9)	
**Mucous membranes**			
Normal	517	7 (1.4)	–
Pale	12	0	
Hyperemic	2	0	
Icteric	1	0	
**Heart murmur**			
Absent	507	0	–
Present	9	6 (66.7)	
**Heart rhythm**			
Regular	514	6 (1.4)	–
Irregular	46	0	
**Lung sounds**			
Normal	430	6 (1.2)	–
Abnormal	72	0	
**Abdominal palpation**			
Normal	487	5 (1.0)	0.57
Organomegaly	19	1 (5.3)	
**Coughing**			
Absent	408	0	–
Present	38	6 (15.8)	
**Vomiting**			
Absent	357	3 (0.8)	0.04
Present	58	3 (5.2)	
**Comorbidity**			
Absent	416	6 (1.4)	0.80
Present	144	1 (0.7)	

*HTW-Ag, Dirofilaria immitis antigen*.

* *Total numbers are presented according to the data capture forms. They may differ from the total number of cats examined (586)*.

** *Too thin–Body condition score 1, 2 and 3; Ideal/Above ideal–Body condition score−4, 5, 6, and 7; Overweight/Obese—Body condition score 8 and 9*.

*A binomial random effects logistic regression (P < 0.05) model was used to identify associations between each of the clinical signs and detection of HTW-Ag*.

Among the HTW-Ag positive cats, information on clinical signs was complete for five cats. These five cats coughed, had a heart murmur with normal heart rhythm, normal abdominal palpation, normal mucous membranes, and normal lung sounds. Among these five cats, two presented with normal BCS, no dyspnea and no vomiting. Two presented with a normal BCS, no dyspnea but had a history of vomiting, and another cat was underweight with dyspnea and vomiting. Among the two cats with incomplete records, one coughed but did not have vomiting and did not allow abdominal palpation nor thoracic auscultation whilst the other had feline gingivitis-stomatitis complex and a heart murmur but lacked any information on the presence of vomiting or coughing.

## Discussion

The overall seroprevalence for HTW-Ag (1.2%), was similar to the results reported before, in the same area, using different inclusion criteria (15), suggesting that HTW is a true threat for the local cat population. Especially when canine HTW infection prevalence in unprotected dogs living in the area is known to range from 7.7 to 38.1% (16). The number of cats exposed to HTW may be even higher since cats were not tested for HTW-Ab, which would detect those cats previously exposed but not harboring adult worms (17). Feline HTW is known to be a disease that many veterinarians and cat owners are not aware of (18). That may be taken as true considering that in this study only 12.1% of the cats had a history of chemoprophylaxis.

Although retroviral infections are known to increase the susceptibility of cats to other pathogens (19), among the 87 retrovirus infected cats reported in this study, none tested HTW-Ag positive, suggesting that neither FIV nor FeLV enhance the risk of cats becoming HTW infected as reported in a previous study (5).

HTW-Ag seropositivity was associated with vomiting in three cats in the present study, an observation that has been described, although not explained, in previous studies of heartworm disease sign in cats (9, 11).

One HTW-Ag positive cat was reported to live entirely indoors. Cats are attractive to the endophilic, broad-ranging vector (eclectic) *Culex quinquefasciatus* (20, 21), however all infected cats were from the suburban area where the conserved natural areas keep humidity high and land occupation low, which enhance sylvatic mosquito species (e.g., *Ochlerotatus scapularis, Ochlerotatus taeniorhynchus*) density but not the *Cx. quinquefasciatus* (22). Although the enhanced mosquito species population in conserved natural areas seems to lack importance in transmitting heartworm to cats, they are important vectors to dogs (21). Since infected dogs are more attractive to mosquitoes than the uninfected, large numbers of microfilaremic dogs in an area will enhance the chance for mosquitoes to carry the parasite (23, 24). Therefore, *Cx. quinquefasciatus* may seek those dogs for blood-feeding, acquire the L1, and transmit the infective L3 to susceptible cats during a subsequent bloodmeal.

In this study we confirmed that feline HTW infection was present in Rio de Janeiro. Therefore, the local veterinarians should include HTW in the differential diagnosis whenever a cat presents with asthma-like signs, unexplained vomiting (10), and heart murmur. Since at this point in time HTW-Ag or HTW-Ab testing cannot be performed because there is no test available in the Brazilian marketplace, veterinarians can only perform chest x-rays and echocardiography in an attempt to confirm the infection (6, 25).

Veterinarians must keep in mind that those infections are insidious and life threatening, therefore they must prioritize educating their clients, testing their patients whenever possible and promoting prophylaxis.

## Conclusions

Cats are at risk of HTW infection in the high canine prevalence area of Rio de Janeiro. No risk factor evaluated was shown to enhance the likelihood for infection with *D. immitis* suggesting that all cats are at risk. Since HTW infection can be life-threatening and treatment options are limited, these results reinforce the need for veterinarians to enhance education of cat caretakers and encourage compliance with recommended prophylaxis procedures.

## Data Availability Statement

The raw data supporting the conclusions of this article will be made available by the authors, without undue reservation.

## Ethics Statement

The animal study was reviewed and approved by Comissão de Ética do Uso de Animais (Institutional Animal Care and Use Committee) of the Instituto de Veterinária—Universidade Federal Rural do Rio de Janeiro, under the Approval Number: 6004310720. Written informed consent was obtained from the owners for the participation of their animals in this study.

## Author Contributions

BA, FM-d-A, and NL designed the study, analyzed the data, and drafted the manuscript. LA, JP, FK, and JL performed statistical analysis, participated in data analysis, and helped draft the manuscript. BA, DC, ASB, AB, RC, and RB examined the cats, obtained blood samples, and participated in data analysis. BA, DC, ASB, AB, NL, and FM-d-A analyzed the samples. All authors have read and approved the final manuscript.

## Funding

IDEXX Laboratories, Westbrook, ME, USA financed the publication fees.

## Conflict of Interest

NL is a consultant for Boehringer Ingelheim, IDEXX, and Zoetis in Brazil. JL is a consultant and research collaborator with IDEXX and Zoetis. The remaining authors declare that the research was conducted in the absence of any commercial or financial relationships that could be construed as a potential conflict of interest.

## Publisher's Note

All claims expressed in this article are solely those of the authors and do not necessarily represent those of their affiliated organizations, or those of the publisher, the editors and the reviewers. Any product that may be evaluated in this article, or claim that may be made by its manufacturer, is not guaranteed or endorsed by the publisher.
