# Supplementary material for: Feline Heartworm in Clinical Settings in a High Canine Prevalence Area
Source: Front Vet Sci. 2022 Feb 10;9:819082. doi: 10.3389/fvets.2022.819082 (PMC8866439; doi:10.3389/fvets.2022.819082)
Supplement: Supplementary file 1 [file Data_Sheet_1.PDF]

Sample number:____________

DATA CAPTURE FORM

DATE: _____/_____/______ **VETERINARIAN**: ____

LOCAL OF PRESENTATION: ________________________________

OWNER'S NAME: ___________________________ **PHONE**: _____________________________

PATIENT

**NAME**	______________________	**AGE:**		**BREED:**		**Sex**: ( )M ( )F	
**REPRODUCTIVE STATUS:**	( )	Intact	( )	Neutered	( )	Pregnant	
**LIFE STYLE**:	( )	Indoors only	( )	Indoors/ outdoors	( )	Outdoors	
**COLLABORATE:**	( )	Yes	( )	No			
**COHABITES**	( )	Yes	How many?	Dogs_______	Cats_______	( )	No
**Neighborhood:**	____						

PHYSICAL EXAM

**BODY CONDITION SCORE**	( ) 1 , 2 or 3	( ) 4, 5 ,6 or 7	( ) 8 or 9	
**HEARTWORM PREVENTION**:	( ) Yes	Product?___________	( ) Doesn't know	( ) No
**REGULAR DEWORMING**	( ) Yes	Product?___________	( ) Doesn't know	( ) No
**RECEIVED FeLV VACCINE?**	( ) Yes		( ) Doesn't know	( ) No
**COMORBIDITY?**	( ) Yes	Which? ___________	( ) Doesn't know	( ) No
**VOMITING:**	( ) Yes		( ) Doesn't know	( ) No
**COUGHING:**	( ) Yes		( ) Doesn't know	( ) No
**DYSPNEA**:	( ) Yes			( ) No

**MUCOUS MEMBRANES**:	( ) Normal	( ) Pale	( ) Icterus	( ) Cyanotic
	( ) Congested		( ) Not observed	
**ABDOMINAL PALPATION**:	( ) Normal	( ) Abnormal	( ) Not observed	
**PULMONARY AUSCULTATION**:	( ) Normal	( ) Abnormal	( ) Not observed	
	( ) Discontinuous noise		( ) Continuous noise	( ) Muffled
**HEART AUSCULTATION:** **Murmur**	( ) Yes	( ) No	( ) Not observed	
**Rhythm**	( ) Regular	( ) Irregular		

Observations:

____
____
____
____
